# The social value of investing in public health across the life course: a systematic scoping review

**DOI:** 10.1186/s12889-020-08685-7

**Published:** 2020-05-01

**Authors:** Kathryn Ashton, Peter Schröder-Bäck, Timo Clemens, Mariana Dyakova, Anna Stielke, Mark A. Bellis

**Affiliations:** 1 Policy and International Health, WHO Collaborating Centre on Investment for Health and Well-being, Public Health Wales, Capital Quarter 2, Tyndall Street, Cardiff, CF104BZ Wales; 2 Department of International Health, Faculty of Health, Medicine and Life Sciences, School CAPHRI (Care and Public Health Research Institute), Duboisdomein 30, 6229 GT Maastricht, The Netherlands

**Keywords:** Social value, Public health, Social return on investment, social cost-benefit analysis, life course

## Abstract

**Background:**

Making the case for investing in public health by illustrating the social, economic and environmental value of public health interventions is imperative. Economic methodologies to help capture the social value of public health interventions such as Social Return on Investment (SROI) and Social Cost-Benefit Analysis (SCBA) have been developed over past decades. The life course approach in public health reinforces the importance of investment to ensure a good start in life to safeguarding a safe, healthy and active older age. This novel review maps an overview of the application of SROI and SCBA in the existing literature to identify the social value of public health interventions at individual stages of the life course.

**Methods:**

A systematic scoping review was conducted on peer-reviewed and grey literature to identify SROI and SCBA studies of public health interventions published between January 1996 and June 2019. All primary research articles published in the English language from high-income countries that presented SROI and SCBA outputs were included. Studies were mapped into stages of the life course, and data on the characteristics of the studies were extracted to help understand the application of social value methodology to assess the value of public health interventions.

**Results:**

Overall 40 SROI studies were included in the final data extraction, of which 37 were published in the grey literature. No SCBA studies were identified in the search. Evidence was detected at each stage of the life course which included; the birth, neonatal period, postnatal period and infancy (*n* = 2); childhood and adolescence (*n* = 17); adulthood (main employment and reproductive years) (*n* = 8); and older adulthood (*n* = 6). In addition, 7 studies were identified as cross-cutting across the life course in their aims.

**Conclusion:**

This review contributes to the growing evidence base that demonstrates the use of social value methodologies within the field of public health. By mapping evidence across stages of the life course, this study can be used as a starting point by public health professionals and institutions to take forward current thinking about moving away from traditional economic measures, to capturing social value when investing in interventions across the life course.

## Background

The need for investment in health and well-being is stronger than ever in the face of multiple challenges and adversities [1]. This is becoming of particular importance as countries are moving away from traditional methods of measuring success (for example, analysing Gross Domestic Product (GDP)) towards measuring wider economic and social value created. For example, in 2019, the New Zealand Government introduced a ‘wellbeing budget’ and have broadened their definition of success to incorporate not only the health of their finances, but also of their natural resources, people and communities [2]. Making the case for investing in public health by collectively illustrating the social, economic and environmental value of public health interventions is imperative to enabling sustainable and fair policy and action for the benefit of people, communities and societies.

Historically, traditional ‘value for money’ approaches such as cost-effectiveness and cost-utility have been the overriding factor which has determined all public sector procurement decisions, taking into account only the monetarised costs of productivity and outputs of an intervention. This is underpinned by a broad evidence base illustrating the return on investment in economic terms and value for money of investing in public health interventions across the life course [1, 3, 4]. However, due to the potential added value of public health interventions (social and environmental, as well as physical) on an individual’s health and well-being, it is becoming increasingly important to capture the wider social value of interventions, services and policies [5, 6].

Social value is defined as the quantification of the relative importance that people place on the changes they experience in their lives [7] accounting for the broader human and societal factors that result from an intervention. For example, the value individuals experience from increasing their confidence, or from living next to a park in a community. Investing in something which creates social value goes beyond the financial value of the service being delivered, to include potential benefits to the local and national economy, the individuals involved, their families and communities.

By moving away from traditional measures of capturing financial value, social value measurements present the full holistic range of outcomes, which is imperative to establishing impact and providing an enhanced understanding of reality [3]. Internationally, there is a body of evidence which uses health economic measurement techniques that capture the social value of investing in public health [1, 8–10]. For example, the impact on inequalities, local employment, health and well-being, community development, social capital and environmental sustainability. Social Cost-Benefit Analysis (SCBA) and Social Return on Investment (SROI) are the predominant tools used to assess the wider value of services or interventions by identifying and evaluating ‘soft’ outcomes, which have traditionally been difficult to measure [8]. SCBA places a monetary value on predetermined outcomes not conventionally measured by other economic methods, such as the well-being of individuals and wider stakeholders such as family or the community. SROI takes this another step further and consists of a framework for measuring a much broader concept of value by measuring change that matters to stakeholders, including a consideration of the economic, social and environmental impacts of investments [11]. Carried out either retrospectively (evaluative) or prospectively (forecast), SROI can help organisations move away from purely financial accounting towards a more comprehensive accountability of value created through an inclusive process of stakeholder engagement and involvement [3, 11].

A vast body of evidence illustrates that key stages across people’s lives have particular significance to their health and well-being, which is reflected through the life course approach in public health [12–14]. A life course approach suggests that an individual’s health, a population’s chronic disease epidemiology and health equity is dependent on the interaction of multiple risk factors, all apparent at different phases across people’s lives [1, 14–16]. Across an individual’s life, biological, social and environmental influences can accumulate and have positive and negative effects on the conditions for mental and physical health [13]. Examples are the associations between family influence and childhood obesity, or the socioeconomic characteristics of the mother’s country of birth and psychotropic medication in Swedish adolescents [14, 17]. The life course approach reinforces the importance of strong investment from ensuring a good start in life to safeguarding a safe, healthy and active older age. By addressing not only the consequences of ill health, but considering the causes and contributors, the life course approach promotes timely investments which produce a high rate of return for both the health of the public, but also financial benefits to the economy [18].

The life course can be split into the following key stages: 1) birth, neonatal period and infancy; 2) early and later childhood and adolescence; 3) adulthood (main employment and reproductive early years); and 4) older adulthood [19]. By investing at each stage, evidence suggests societal and economic benefits can be achieved, as well as improvements in health at the individual level [12]. The case for investment in the early years has been evidenced through international research [3], and promoted through high profile reports, such as the Marmot Review [13] and the World Health Organization’s Commission on Social Determinants of Health [20]. Giving every child the best start in life is crucial to reducing health inequalities and inequity. The early childhood period is considered to be the most important developmental phase throughout the life course [21], and harmful childhood experiences are linked to long-lasting disadvantage and ill health, with substantive costs to the individual and the economy [22]. For example, it has been estimated that investing in breastfeeding has a clear positive return on investment across the life course [23]. In addition, poor education can be detrimental to health and life prospects [24, 25], with evidence suggesting that investing in early education can result in high social and economic returns, and also has positive intergenerational effects [1]. After childhood, adult life involves maintaining the highest possible level of function. The rate of decline at this stage is largely determined by behavioural lifestyle factors adopted at this stage, or previously, such as smoking, alcohol consumption, levels of physical activity and diet. Finally, the importance of investing in health in older life is focussed on preventing disability and maintaining independence [26].

Previous secondary research has been undertaken to collate existing evidence on the SROI of public health interventions [3, 9, 10]. The review outlined in this paper aims to build on these findings to map out the existing SROI and SCBA evidence on the social value of public health interventions across stages of the life course. By exploring the extent of the literature, this review will identify the characteristics of SROI and SCBA evidence of public health interventions, illustrate how evidence is distributed across stages of the life course, outline the range of SROI values presented in this evidence, and suggest what gaps exist in the current evidence base at the different life course stages.

## Methods

To gain an overarching understanding of the available evidence on the social value of public health interventions across the life course, a systematic scoping review was undertaken, using a comprehensive search strategy and selection criteria. A scoping review is defined as a preliminary assessment of the potential size and scope of the available research literature, which aims to identify the nature and extent of research evidence on a topic. Evidence suggests that used appropriately, this method can apply a comprehensive and systematic approach to mapping the literature, key concepts, theories, evidence and research gaps in a field using broadly framed questions [27].

### Search strategy

Evidence was collated from peer-reviewed academic research and grey literature. The search terms used were “public health” OR “health promotion” OR “primary prevention” OR “life course” OR “health” and “interven*” or “program*” and “social return on investment” OR “social cost benefit analysis”. These search terms were used to search on title or abstract within peer-reviewed databases (PubMed and ProQuest). The grey literature was explored using the same search terms as the academic search on Google Scholar and organisational websites (World Health Organization, public health institutional websites, Social Value UK and the New Economic Foundation). Manual snowball and forward citation searches were also conducted on the academic and grey literature identified for inclusion. One researcher independently conducted the search in July 2019. An additional researcher also screened the evidence, and any conflicts in opinion were discussed by the two researchers and a consensus agreed upon.

### Inclusion and exclusion criteria

At the initial search stage, publications were included if they were written in the English language and published from January 1996, as this was when the first social value study using SROI was published, to June 2019. At the screening stage, publications were only included if they focussed specifically on SCBA, SROI or social value of public health interventions, and included the SROI output of primary studies from high-income countries to further limit the studies included. Finally, at the eligibility stage, articles were excluded if they were solely protocol papers and included no data or description of the economic, social or environmental returns of a public health intervention.

### Data extraction and synthesis

For the purpose of this study, all evidence captured was categorised into the stages of the life course; birth, neonatal period, post-natal period and infancy, childhood and adolescence, adulthood (main employment and reproductive years), and older adulthood. Family interventions targeted at developing the health and well-being of children were included in the ‘Childhood and adolescence’ category, as the primary aims were to provide support for the children. An additional category of ‘Cross-cutting’ was also included to capture those interventions targeted at populations which cut across several stages of the life course.

A summary table was used to capture necessary information about each individual study. This included year and country of publication, social value methodology used, who commissioned the study, public health topic the intervention was focussed on, target population of the intervention being assessed, details of stakeholder engagement, how outcomes were measured, economic results including the crude SROI ratio for the time horizons included in the study, type of publication (academic or grey literature) and limitations of the study identified by the authors. In addition, to assess the quality of the identified studies, a quality assessment framework based on a 0–12 point scoring index [28] as used in similar studies [9], was used to score the evidence which contributed to the understanding of the use of SROI and SCBA methodology. This framework assesses the quality of studies based on the following criteria: transparency about why SROI was chosen; documentation of the analysis; study design including approximation of counterfactual; precision of the analysis; and reflection of the results.

The information extracted was used to develop a literature map that helped to illustrate the distribution of the evidence of the social value of investing in public health across the stages of the life course. Public health topics, target populations, aims of intervention and the crude SROI ratios were summarised for each stage of the life course. Finally, the summaries presented were used to suggest gaps in the existing evidence base.

## Results

**Fig. 1:**
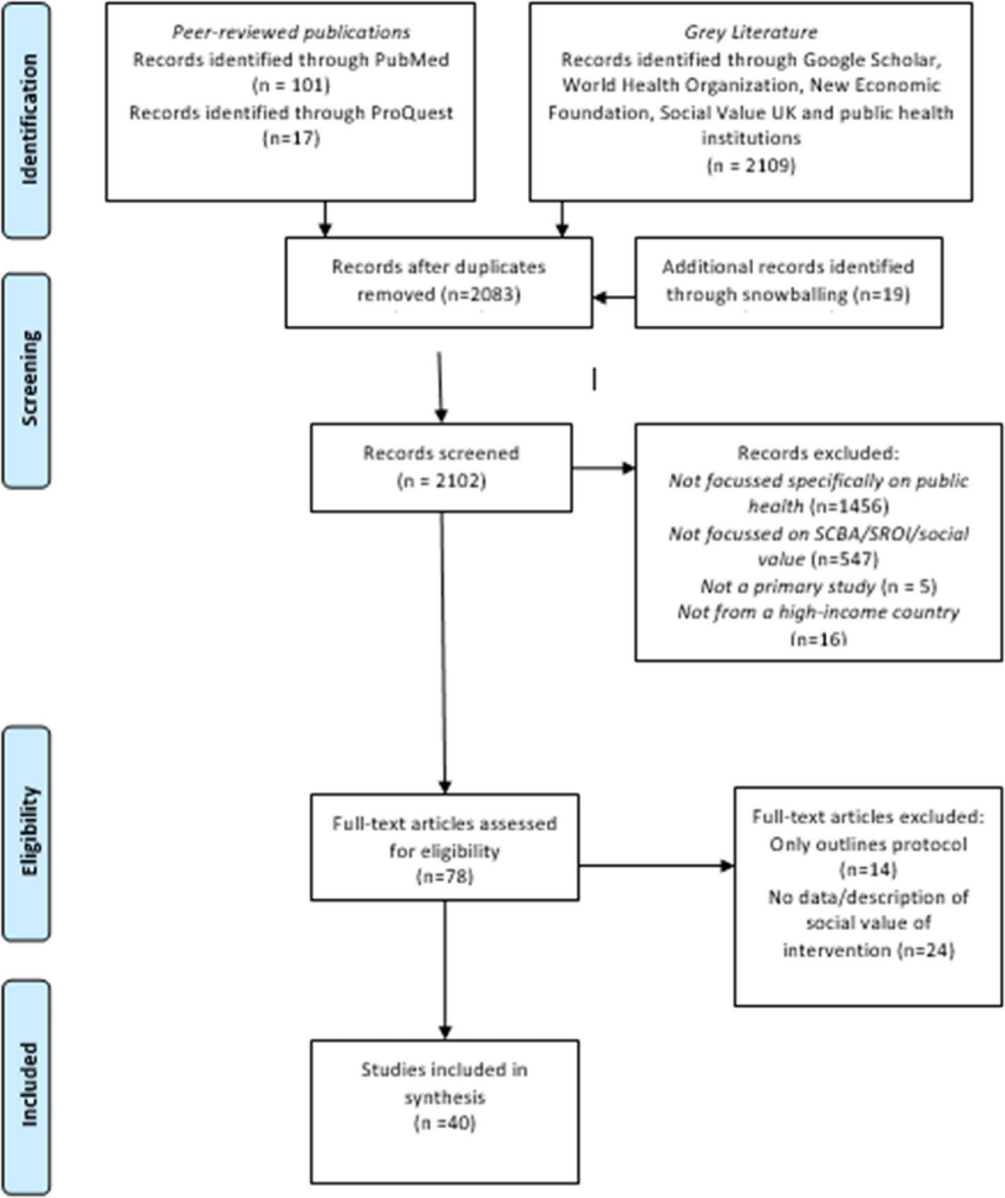
PRISMA flowchart

To report the findings of this scoping review, the Preferred Reporting Items for Systematic Review and Meta-Analyses (PRISMA) approach was followed [29]; Fig. 1]. Following a systematic approach, a total of 40 studies were identified for inclusion in the final evidence synthesis.

### Study characteristics

Of the 40 included studies, only three were published in the academic literature with the remaining 37 published in the grey literature. With regards to country of origin for the studies, 87.5% (*n* = 35) had been carried out in United Kingdom, with the remaining 12.5% (*n* = 5) originating from Ireland, Australia, New Zealand, Canada and the Netherlands. Although the search strategy used in this study included SCBA studies, only SROI studies were identified in the literature. Of these, seven were categorised as prospective or forecast SROI studies (i.e. predicted the impact of a project or activity), with the remainder being evaluative or retrospective SROIs (i.e. measures the change a project of activity has delivered). The evidence identified through this review indicates that the number of SROI studies peaked in 2012 and 2013, with a steady decline towards 2019 (Table 1).

**Table 1:** Study characteristics of included studies by stages of the life course

Study characteristics		Stage of the life course (n)				
	Birth, neonatal period, postnatal period and infancy	Childhood and adolescence	Adulthood (main employment and reproductive years)	Older adulthood	Cross-cutting	Total
**Source of publication**						
Academic	1	0	0	2	0	3
Grey	1	17	8	4	7	37
**Country**						
United Kingdom	1	15	7	6	7	35
Ireland	1	0	0	0	0	1
Australia	0	1	0	0	0	1
New Zealand	0	1	0	0	0	1
Canada	0	0	0	0	0	1
The Netherlands	0	0	1	0	0	1
**Year published**						
2019	1	1	0	1	0	3
2018	0	1	0	1	1	3
2017	0	1	1	0	0	2
2016	0	0	0	0	2	2
2015	0	0	0	1	0	1
2014	0	2	1	1	0	4
2013	1	2	2	0	1	6
2012	0	3	1	1	1	6
2011	0	1	0	1	2	4
2010	0	2	1	0	0	3
2009–2007	0	4	2	0	0	6
**Social value methodology**						
SROI^a^	2	17	8	6	7	40
SCBA^b^	0	0	0	0	0	0
**Type of SROI**						
Forecast/prospective	0	4	2	0	1	7
Evaluative/retrospective	2	13	6	6	6	33
**Quality scores**						
High quality (score > =7)	2	17	4	5	7	36
Low quality (score < 7)	0	0	3	1	0	4
**Total**	2	17	8	6	7	40

^a^SROI Social Return on Investment

^b^SCBA Social Cost Benefit Analysis

With regards to the quality of the final included evidence, quality scores for individual studies ranged from 4 to 11 (mean = 8.45). Using the benchmark of a score of seven or above to indicate high quality [28], 36 studies (90%) were considered to be of a high quality with 4 considered to be of a lower quality using the information within the publications (Table 1). No study achieved a maximum score of 12 which reflects findings elsewhere [19] and is because none of the SROI analyses identified in this review had a control group within their SROI designs, which is an element within the scoring index of the quality assessment framework used in this review [28].

### Distribution of social value evidence across stages of the life course

**Fig. 2:**
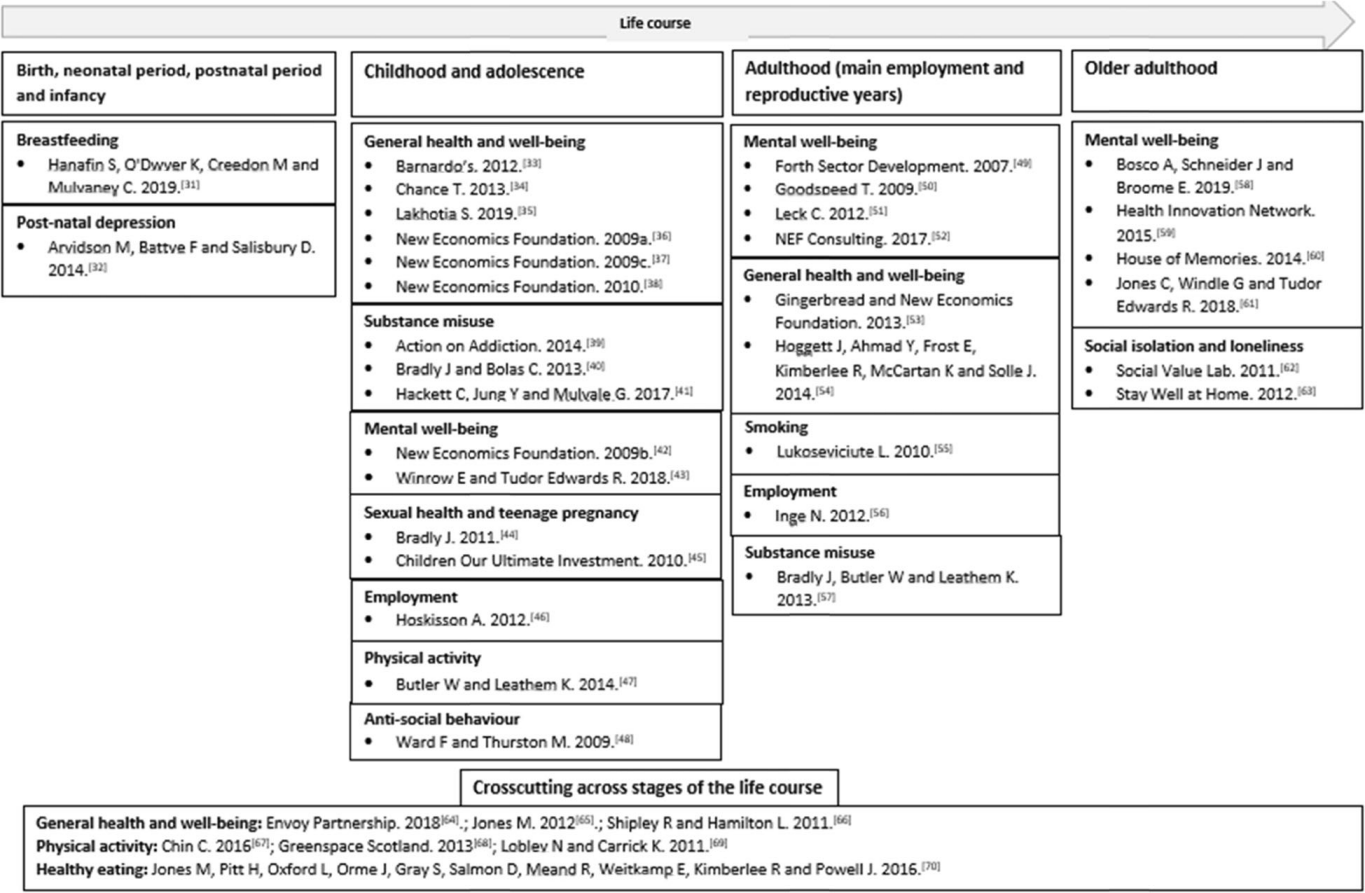
Literature Map: Evidence for Social Return on Investment (SROI) across stages of the life course

The literature map (Fig. 2) illustrates the evidence included in this scoping review, according to life course stage and public health topic. Within the first stage of the life course, which we classified as ‘birth, neonatal period, postnatal period and infancy’, two studies were identified. A total of 16 studies were identified at the next stage of the life course categorised as ‘Childhood and adolescence’, followed by nine studies at the ‘Adulthood (main employment and reproductive years) stage. Finally, six studies were categorised into the ‘Older adulthood’ stage. In addition, there were seven studies which were included into the supplementary category of ‘Crosscutting’ as these interventions were targeted at a range of individuals at differing stages of the life course, for example an intervention which developed volunteer Community Champions to promote health and well-being to all residents in a local community in England [30].

### Birth, neonatal period, postnatal period and infancy

Of the two studies identified in the first stage of the life course, one focussed on the topic of breastfeeding with mother [31] reporting a crude SROI of €15.85 per €1 invested, whilst the other outlined the SROI of an intervention to support those affected by post-natal depression [32] showing a crude SROI of £6.50 per £1 invested [Table 2].

**Table 2:** Social return on investment (SROI) of public health interventions: birth, neonatal period, postnatal period and infancy

Reference	Public health topic	Country	Population	Aim of Intervention	Crude SROI ratio for assessed time horizon	Quality score
Hanafin et al. 2019 [31]	Breastfeeding	Ireland	Mothers	Groups aimed to provide support, knowledge and advice to breastfeeding mothers and through that to improve maternal confidence and capacity to breastfeed.	€15.85/€1 invested	8
Arvidson, Battye and Salisbury. 2013 [32]	Post-natal depression	England	Families affected by post-natal depression (PND)	To provide high-quality community-based support to those affected by PND. To raise awareness of PND amongst health professionals. To recruit and train local people to provide volunteer-led support services.	£6.50/£1 invested	7

### Childhood and adolescence

In total, 16 SROI studies were identified in the childhood and adolescence stage of the life course (Table 3). These focussed on a range of public health topics which included the following: general health and well-being [33–38], substance misuse [39–41], mental well-being [42, 43], sexual health and teenage pregnancy [44, 45],, employment [46], physical activity [47] and anti-social behaviour [48]. SROI ratios for interventions at this stage of the life course ranged from £2 per £1 invested [33], to £9.20 per £1 invested [37].

**Table 3:** Social return on investment (SROI) of public health interventions: childhood and adolescence

Reference	Public health topic	Country	Population	Aim of Intervention	Crude SROI ratio for assessed time horizon	Quality score
Barnardo’s 2012 [33]	General health and well-being	England	Families with young children who need additional support/have behavioural needs	Numerous aims to improve the health and well-being of children and their families through services such as ‘Stay and Play’, ‘Family Support Workers’, ‘Tiny Toes’ and the ‘Triple P Parenting Programme’.	Stay and Play £2/£1 invested Family Support Workers £4.50/£1 invested Tiny Toes £3.50/£1 invested Triple P £2.50/£1 invested	10
Chance 2013 [34]	General health and well-being	England	Disadvantaged two year olds accessing childcare and their immediate families	To improve outcomes for children and narrow the gap in educational achievement between them and other children.	£8.40/£1 invested	9
Lakhotia 2019 [35]	General health and well-being	New Zealand	Families with children aged 3–8 years	Improving parental capabilities to promote emotional and social competence in children and to prevent, reduce and treat conduct problems.	NZ$3.75:NZ$1 invested	11
New Economics Foundation 2009a [36]	General health and well-being	Wales	Young people and children (from about 5 to 14) and their families	Preventative early intervention service for young people and children (from about 5 to 14) and their families, where there are recently emerging emotional, behavioural or mental health issues.	£7.60/£1 invested	9
New Economics Foundation 2009c [37]	General health and well-being	England	Children and families in the local area	Provides short-term, focussed and flexible support for children, young people and families in crisis.	£9.20/£1 invested	9
New Economics Foundation 2010 [38]	General health and well-being	England	Vulnerable families	To work with the most vulnerable families to treat the cause and effects of multiple issues, such as domestic violence and anti-social behaviour.	£4.28/£1 invested	10
Action on Addiction 2014 [39]	Substance misuse	England	Children and young people aged 8–17 years	Supports children and young people aged 8–17 who are experiencing the effects of substance misuse within the family. Programme offers a ‘whole family approach’.	£2.76/£1 invested	7
Bradly and Bolas 2013 [40]	Substance misuse	England	Individuals aged 16–19 years	To deliver a psycho-social intervention alongside harm reduction work.	£3.91/£1 invested	10
Hackett, Jung and Mulvale 2017 [41]	Substance misuse	Canada	Individuals aged 13–19 years	Provides treatment to young people between the ages of 13–19 who face addiction issues, as well as behavioural and psycho-social challenges.	£7/£1 invested	9
New Economics Foundation 2009b [42]	Mental well-being	England	Children in the local area	To provide universal services to children in the area.	£4.60/£1 invested	10
Winrow and Tudor Edwards 2018 [43]	Mental well-being	Wales	Primary school age children	Delivers music sessions in two schools in disadvantaged neighbourhoods based on the El Sistema method - aims to improve educational and well-being outcomes for children who face socio-economic challenges	£6.69/£1 invested	9
Bradly 2011 [44]	Sexual health	England	Adolescents	To support the delivery of sexual health services for young people in community settings that they would typically access e.g. school, youth services.	£8.75/£1 invested	11
Children Our Ultimate Investment 2010 [45]	Sexual health	England	At-risk young people referred to the programme by their school	To provide at-risk young people of both sexes with real life experience of mentoring and caring for a small child by enrolling in a 15–20 week course for one afternoon a week where the teenager is paired with a child in a nursery.	£5.52/£1 invested	8
Hoskisson 2012 [46]	Employment	Australia	Young offenders	To create meaningful employment opportunities and offer education, training and workplace mentoring for young offenders.	£2.33/£1 invested	9
Butler and Leathem 2014 [47]	Physical activity	England	Young people aged 10–19 years	To increase the range of sporting opportunity, to ensure that local delivery reflects needs, multigenerational and build evidence base to show sport as an instrument of social change.	£4.21/£1 invested	9
Ward and Thurston 2009 [48]	Anti-social behaviour	England	Young people aged 11–16 years	Remotivate young people aged 11–16 years who were disaffected and/or displaying antisocial behaviour.	£3.70/£1 invested	9

### Adulthood (main employment and reproductive years)

Of the nine studies identified within the adulthood stage of the life course, four focussed on interventions that aimed to improve mental well-being [49–52], two on general health and well-being interventions [53, 54], one on smoking [55] one on employment [56] and one on substance misuse [57] (Table 4). The SROI ratios ranged from £0.66 per £1 invested reported for an intervention seeking new ways of working with troubled families by changing trajectories for families and changing the ways services are delivered to them [54], to £7 per £1 invested for an intervention that focussed on providing support for adults with multiple long-term health conditions, low-level emotional health concerns or lifestyle or social issues [52].

**Table 4:** Social return on investment (SROI) of public health interventions: Adulthood (main employment and reproductive years)

Reference	Public health topic	Country	Population	Aim of Intervention	Crude SROI ratio for assessed time horizon	Quality score
Forth Sector Development 2007 [49]	Mental well-being	Scotland	Individuals with mental health problems who have recently become unemployed	To support people with mental health problems after they have become unemployed to improve their overall health and well-being.	£1.57/£1 invested	8
Goodspeed 2009 [50]	Mental well-being	England	Individuals with mental health problems	To successfully develop and train people with mental health problems in a genuine business environment to encourage independence and self-esteem through work.	£3.09/£1 invested	9
Leck 2012 [51]	Mental well-being	England	Aged between 14 and 65 with varying levels of learning difficulties/disabilities, mental health issues and acquired brain injuries, and young people who are struggling in mainstream education	To provide therapy, education, training, work and friendship for people with a wide range of individual needs	£3.77/£1 invested	9
New Economics Foundation 2017 [52]	Mental well-being	England	Adults with multiple long term health conditions, low-level emotional health concerns, or lifestyles of social issues	To provide support for adults with multiple long-term health conditions, low-level emotional health concerns, or lifestyle or social issues	£7/£1 invested	0
Gingerbread and New Economics Foundation 2013 [53]	General health and well-being	Wales	Single parent families	To provide opportunities for single parent families to get together, meet new people and share experiences.	£4.27/£1 invested	9
Hoggett et al 2014 [54]	General health and well-being	England	Troubled families	To seek new ways of working with troubled families - changing trajectories for families and changing the ways services are delivered to them.	£0.66/£1 invested	6
Lukoseviciute 2010 [55]	Smoking	Netherlands	Individuals aged 20–65+ years	To encourage individuals to quit smoking through a smoking cessation programme	€2.2/€1 invested	4
Inge 2012 [56]	Employment	England and Wales	Disadvantaged young people (particularly those who have been homeless)	Ready for Work is a Business in the Community (BITC) programme that engages business to support disadvantaged groups, particularly people who have experienced homelessness, into employment.	£3.12/£1 invested	10
Bradly, Butler and Leathem 2013 [57]	Substance misuse	England	Adults recovering from drug misuse	To promote recovery and community integration for people who have experienced problematic drug and alcohol use -aftercare.	£4.02/£1 invested	9

### Older adulthood

The six studies identified in this review within the life course stage of older adulthood focussed on two main public health topics; mental well-being [58–61] and social isolation and loneliness [62, 63] (Table 5). The SROI ratios ranged from £11 per £1 [63] to £1.20 per £1 invested [58].

**Table 5:** Social return on investment (SROI) of public health interventions: Older adulthood

Reference	Public health topic	Country	Population	Intervention	Crude SROI ratio for assessed time horizon	Quality score
Bosco et al 2019 [58]	Mental well-being	England	Residents of residential care homes, with and without dementia	To experiment with the delivery of high-quality arts interventions in care homes, to understand the impact on quality of life, mental well-being and general health of stakeholders.	£1.20/£1 invested	10
Health Innovation Network 2015 [59]	Mental well-being	England	Individuals with dementia, carers, families and others associated with the group.	To provide peer support for people with dementia through providing a facilitated environment for people to meet and socialise, with a variety of dementia appropriate activities to engage group members.	Ranged from £1.17 - £5.18/£1 invested dependent on design and structure of the group	7
House of Memories 2014 [60]	Mental well-being	England	Residential carers	To provide health, social care and housing workforce with practical skills and resources to help people live well with dementia.	£8.66/£1 invested	9
Jones, Windle and Tudor Edwards 2018 [61]	Mental well-being	England and Wales	Individuals living with dementia	A visual arts programme for people living with dementia to improve their mental health and well-being.	£5.18/£1 invested	10
Social Value Lab 2011 [62]	Social isolation and loneliness	England	Elderly population	To reduce the isolation and loneliness experienced by older people, to enable them to make positive lifestyle changes.	£8.27/£1 invested	10
Stay Well at Home 2012 [63]	Social isolation and loneliness	England	Older people at risk of losing their independence	To help older people maintain their independence and improve their quality of life.	£11/£1 invested	5

### Cross-cutting across the life course

Seven SROI studies were classified as aiming to cut across different stages of the life course, with three aimed at promoting general health and well-being [30, 64, 65], three focussing on interventions that aimed to improve physical activity [66–68], and one focussed on healthy eating [69] (Table 6). For interventions that cut across the life course, SROI ratios ranged from £44.56 per £1 invested and £2.56 per £1 invested.

**Table 6:** Social return on investment (SROI) of public health interventions: Cross-cutting across the life course

Reference	Public health topic	Country	Population	Aim of Intervention	Crude SROI ratio for assessed time horizon	Quality score
Envoy 2018 [30]	General health and well-being	England	Individuals in local communities	Community Champions volunteer at a community centre/hub to promote the health and well-being of all residents.	£5/£1 invested	10
Jones 2012 [64]	General health and well-being	England	People vulnerable to poor health through weight-related issues	To promote healthier lifestyles for people vulnerable to poor health through weight-related issues. The services offer one-to-one and group tailored interventions to help people put into action their lifestyle goals.	£5.42/£1 invested	11
Shipley and Hamilton 2011 [65]	General health and well-being	England	Individuals attending courses which focus on leading healthier lifestyles	To increase the knowledge, understanding, awareness and information about health issues for those people who attend courses which focus on leading healthier lifestyles.	£2.56/£1 invested	10
Chin 2016 [66]	Physical activity	Wales	Disabled individuals referred by their GPs	To improve the health and well-being by increasing the number of disabled people who are physically active.	£44.56/£1 invested	9
Greenspace Scotland 2013 [67]	Physical activity	Scotland	Hospital in-patients, people with learning disabilities, members of ethnic minorities and individuals referred by medical practitioners	Develop and promote walking opportunities across Glasgow, targeting groups least likely to take regular exercise, in order to increase physical activity levels and improve the health and well-being of city residents	£8/£1 invested	7
Lobley and Carrick 2011 [68]	Physical activity	Scotland	General population	To encourage healthier and more physically active lifestyles addressing issues surrounding mental health, physical health and social well-being	£4.40/£1 invested	10
Jones et al 2016 [69]	Healthy eating	England	General population	To promote a good food culture through supporting practical delivery and influencing delivery making	£4.41/£1 invested	11

## Discussion

This review contributes to the growing evidence base that demonstrates the use of social value methodology within the field of public health [9, 10]. It is acknowledged there may be wider existing economic evidence of wider policy interventions assessing potential health benefits, for example of transport and housing policy, however this search focussed on public health interventions directly targeted at improving health. Complementing previous reviews that have aimed to capture the existing evidence on the use of SROI methodology on public health interventions and services [9, 10], this review takes a unique approach to mapping studies from both the academic and grey literature across stages of the life course. Results can be used as a starting point by public health professionals and institutions to develop an understanding of the social value of public health interventions across different stages of the life course, which could be used to inform policy, practice and investment decisions.

This review identified that the majority of SROI studies on public health interventions have been carried out in the United Kingdom. This may be a reflection of the introduction of the Public Services (Social Value) Act 2012 [70] and the growing emphasis to undertake impact assessments, particularly within the private and third sector [71]. Captured evidence was mostly evaluative in nature, with the reporting of SROIs peaking in the years of 2012 and 2013, illustrating a steady decline towards 2019. This is interesting to note, due to the growing interest in recent years of moving away from traditional economic measures of success within economies, towards a wellbeing approach and new measures of capturing progress [2, 72]. Sparse literature was identified within the academic evidence base with the majority being published in the grey literature. This again aligns with existing reviews, which note this may be a result of weaknesses in SROI methodology which potentially stifle opportunity for academic publication [9–10; 74]. Another reason for this may be associated with the type of organisations undertaking SROIs, for example not-for-profit and charitable organisations, who may not traditionally focus on academically publishing their work [73]. In addition, although this study sets out to capture both SROI and SCBA evidence, no SCBA studies were found to focus on public health interventions. These results suggest that SCBA is not yet a recognised methodology used to capture the social value of public health interventions and may require further investigation and promotion. In addition, these results suggest that researchers have found the social value methodologies discussed in this paper potentially difficult to adapt to their scenarios, or difficult and labour intensive to undertake.

With regards to the area of public health, it is interesting to note that no evidence was identified that captured the social value of public health interventions outside of the field of health promotion. For example, screening services, vaccination or environmental health initiatives. The reason for this again may be related to the type of organisations currently utilising SROI to undertake economic evaluations, for example third sector organisations as opposed to national public health institutes. Another reason would be the preference of using more ‘established’ health economics methods, such as cost-effectiveness or cost-benefit analysis, due to availability of ‘hard’ clinical outcomes, such as reduction in mortality, morbidity and hospital admissions. However, these traditional methods fail to capture the ‘soft’ outcomes, related to additional benefits (added value) to the individuals, their families, carers, communities, social, physical and economic environment.

The life course perspective in public health emphasises the important role and variability of social, environmental and economic factors play in the development of different health trajectories across the life stages [74]. Within this review, we mapped the evidence of the social value of public health interventions across the life course. The childhood and adolescence stage comprised almost half of the studies identified (*n* = 17), with most focussing on general health and well-being and substance misuse. Only two studies were found to be reported in the birth, neonatal period, postnatal period and infancy. The small number of studies identified in this first stage of the life course could be due to the methodological challenges in SROI of capturing the value of the long-term outcomes. This is referred to as ‘deadweight’ in SROI methodology, or what would have happened anyway, which is more complex to measure and forecast across the life course [75]. In addition, there are complexities with measuring ‘well-becoming’, which focusses on the future, as opposed to ‘well-being’ which focusses on the present [76], particularly if trying to capture the value of an intervention across the whole life course. The remaining evidence was split relatively equally over the remaining stages of the life course, cutting across the topics areas of mental well-being, social isolation, general health and well-being, substance misuse and healthy eating. As with previous research, all SROI evidence reported encouraging SROI ratios, indicating that the interventions identified at each stage of the life course produced a positive overall social value [9]. These examples can be used as a starting point by stakeholders to help guide further work into estimating the social value of public health interventions at different stages of the life course, depending on the public health area or topic. This would potentially help identify the interventions with highest or higher value for each life stage or across all stages; or which public health areas would be most relevant, for example bring the most value, to invest in within each life stage.

### Limitations

Although the methodology used for this review was appropriate for the aims of the research, there are limitations, which are important to note. The search terms used in our systematic scoping review may not have captured every piece of evidence on the social value of public health services and interventions, in particular those public health interventions which may be referred to by another title. An example of this is community engagement interventions, which could potentially have an impact on the health of the public across particular stages of the life course and create social value. This was coupled with the difficulty of searching for social value studies in the grey literature where no dedicated database exists internationally [9].

As part of a credible methodological process [11], the majority of studies in this review carried out sensitivity analyses on their SROIs based on different scenarios and assumptions. It was beyond the scope of this study to interpret the crude SROI values reported and their associated sensitivity analyses. In addition, this paper did not aim to compare interventions or stages of the life course to identify which create the most social value. The SROI ratios created by undertaking the standardised methodology incorporates elements which make the end ratio unique to the intervention being assessed, for example using differing time horizons and subjectivity around the proxy valuation process within SROI [77]. Also, what is important to measure and how is it valued may differ according to life stage [76]. In order to compare these values, additional work would be required to account for the caveats around the possibilities of making these comparisons.

### Recommendations for further research

There is scope for further research to be undertaken which could build on elements outside of the aims and objectives of this study. This review is the first step to capturing and mapping the social value of public health interventions at different stages of the life course. Further research is needed to understand where social value methodology is best suited in relation to measuring value of interventions at the different life stages. There is a clear need for further high-quality SROI and SCBA studies to be undertaken and academically published, particularly focussing on capturing the social value of services outside of the field of health promotion [10]. In addition, although there is initial research which explores how public health organizations measure value [78], further exploratory work is also required to comprehend how social value is being captured at an institutional level to help build the evidence base and inform the efficient allocation of resources across the life course.

## Conclusion

There is a significant interest in measuring and capturing the social value of public health interventions to help guide investment decisions and aid the efficient allocation of resources. This paper builds on existing research to understand the existing evidence base, taking a unique approach to mapping identified SROI and SCBA evidence across stages of the life course. From the early years of childhood to older adulthood, the importance of capturing social value has been highlighted, with existing SROI research indicating the positive value of investing in public health interventions. This research has indicated that although attempts have been made to measure the social value in public health, further research is needed to develop this field. This includes publishing more case studies within the academic literature, and understanding in more detail how SROI can be used to capture long-term outcomes across all stages of the life course. Additional benefit could be found by further exploring the reasons why some researchers are not utilizing these methodologies and publishing results academically to help develop the evidence base. Results highlighted within this work can be used as a starting point by public health professionals, institutions and across sectors to take forward current thinking about moving away from traditional economic measures, towards considering the wider determinants of health and well-being in their valuations, and to capture and quantify the social value resulting from a wider range of policy initiatives.

## Data Availability

Data sharing is not applicable to this article as no datasets were generated or analysed during the current study.

## References

[1] Dyakova M, Hamelmann C, Bellis MA, Besnier E, Grey CNB, Ashton K, Schwappach A and Clar C. 2017. Investment for health and well-being: a review of the social return on investment from public health policies to support implementing the Sustainable Development Goals by building on Health 2020. Health Evidence Network synthesis report 51. [Online]. Available at: http://www.euro.who.int/en/publications/abstracts/investment-for-health-and-well-being-a-review-of-the-social-return-on-investment-from-public-health-policies-to-support-implementing-the-sustainable-development-goals-by-building-on-health-2020-2017 [Accessed 13th Nov, 2019]. 28956895

[2] New Zealand Treasury. 2019. The Wellbeing Budget. [Online]. Available at: https://treasury.govt.nz/sites/default/files/2019-05/b19-wellbeing-budget.pdf [Accessed 13th Nov, 2019].

[3] Tudor-Edwards R, Bryning L and Lloyd-Williams H. 2016. Transforming Young Lives across Wales: the Economic Argument for Investing in the Early Years. [Online]. Available at: https://cheme.bangor.ac.uk/documents/transforming-young-lives/CHEME%20transforming%20Young%20Lives%20Full%20Report%20Eng%20WEB%202.pdf [Accessed 13^th^ Nov, 2019].

[4] Masters R, Anwar E, Collins B, Cookson R, Capewell S (2017). Return on investment of public health interventions: a systematic review. J Epidemiol Community Health.

[5] Shiell A, Hawe P, Gold L. Complex interventions or complex systems? Implications for health economics evaluation. BMJ. 2008;336(1281). 10.1136/bmj.39569.510521.AD. 10.1136/bmj.39569.510521.AD PMC2413333 18535071

[6] Baker C, Courtney P (2015). Conceptualising the societal value of health and wellbeing and developing indicators for assessment. Eur J Public Health.

[7] Social Value UK. 2019. What is Social Value? [Online]. Available at: http://www.socialvalueuk.org/what-is-social-value/ [Accessed 13^th^ Nov, 2019].

[8] New Economics Foundation. 2013. Economics in policy-making 4. Social CBA and SROI. [Online]. Available at: https://www.nefconsulting.com/wp-content/uploads/2014/10/Briefing-on-SROI-and-CBA.pdf [Accessed 13th Nov, 2019].

[9] Banke-Thomas AO, Madaj B, Charles A, van den Broek N. Social Return on Investment (SROI) methodology to account for value for money of public health interventions: a systematic review. BMC Public Health. 2015;15(582). 10.1186/s12889-015-1935-7. 10.1186/s12889-015-1935-7 PMC4477315 26099274

[10] Hutchinson CL, Berndt A, Forsythe D, Gilbert-Hunt S, George S, Ratcliffe J. Valuing the impact of health and social care programs using social return on investment analysis: how have academics advanced the methodology? A systematic review. BMJ Open. 2019:9–e029789. 10.1136/bmjopen-2019-029789. 10.1136/bmjopen-2019-029789 PMC6720245 31446413

[11] The SROI Network. 2012. *A guide to Social Return on Investment*. [Online]. Available at: http://www.socialvalueuk.org/app/uploads/2016/03/The%20Guide%20to%20Social%20Return%20on%20Investment%202015.pdf [Accessed 13^th^ Nov, 2019].

[12] Jacob CM, Baird J, Barker M, Cooper C and Hanson M. 2017. The importance of a life-course approach to health: Chronic disease risk from preconception through adolescence and adulthood. [Online]. Available at: https://www.who.int/life-course/publications/importance-of-life-course-approach-to-health/en/ [Accessed 18^th^ Nov, 2019].

[13] Marmot M. 2010. Fair Society. Healthy Lives. A Strategic Review of Inequalities in England. [Online]. Available at: https://www.local.gov.uk/marmot-review-report-fair-society-healthy-lives [Accessed 13th Nov, 2019].

[14] Pratt BA and Frost LJ. 2017. The life course approach to health: a rapid review of the literature. White paper. [Online]. Available at: https://www.who.int/life-course/publications/life-course-approach-literature-review.pdf?ua=1 [Accessed 13^th^ Nov, 2019].

[15] Braveman P (2014). What is health equity: and how does a life-course approach take us further toward it?. Matern Child Health J.

[16] Ben-Shlomo Y, Kuh D (2002). A life course approach to chronic disease epidemiology: conceptual models, empirical challenges and interdisciplinary perspectives. Int J Epidemiol.

[17] Van Leeuwen W, Nilsson S, Merlo J (2012). Mother’s country of birth and prescription of psychotropic medication in Swedish adolescents: a life course approach. BMJ Open.

[18] World Health Organization. 2019. Life-course approach. [Online]. Available at: http://www.euro.who.int/en/health-topics/Life-stages [Accessed 13^th^ Nov, 2019].

[19] Kuruvilla S, Sadana R, Villar Montesinos E, Beard J, Franz Vasdeki J, Araujo de Carvalho I, Bosco Thomas R, Brunne Drisse M-N, Daelmans B, Goodman T, Koller T, Officer A, Vogel J, Valentine N, Wootton E, Banerjee A, Magar V, Neira M, Okwo Bele JM, Worning AM, Bustreo F (2018). A life-course approach to health: synergy with sustainable development goals. Bull World Health Organ.

[20] Heckman JJ. 2012. Invest in early childhood development: Reduce deficits, strengthen the economy. [Online]. Available at: https://heckmanequation.org/resource/invest-in-early-childhood-development-reduce-deficits-strengthen-the-economy/ [Accessed 13^th^ Nov, 2019].

[21] Commission on Social Determinants of Health. 2019. Closing the gap in a generation. Health equity through action on the social determinants of health. Final Report of the Commission on Social Determinants of Health. [Online]. Available at: http://www.who.int/social_determinants/thecommission/finalreport/en/ [Accessed 13^th^ Nov 2019].

[22] Heckman JJ (2008). Schools, skills, and synapses. Econ Inq.

[23] Bellis MA, Hughes K, Ford K, Ramos Rodriguez G, Sethi D, Passmore J (2019). Life course health consequences and associated annual costs of adverse childhood experiences across Europe and North America: a systematic review and meta-analysis. Lancet Public Health.

[24] Renfrew MJ, Pokhrel S, Quigley M, McCormick F, Fox-Rushby J, Dodds R and Williams A. 2012. Preventing disease and saving resources: the potential contribution of increasing breastfeeding rates in the UK. [Online]. Available at: https://www.unicef.org.uk/wp-content/uploads/sites/2/2012/11/Preventing_disease_saving_resources.pdf. [Accessed 13th Nov, 2019].

[25] Marmot M, Allen J, Bell R, Bloomer E, Goldblatt P (2012). WHO European review of social determinants of health and the health divide. Lancet.

[26] World Health Organization. 2000. The implications of training of embracing. A life course approach to health [Online]. Available at: https://www.who.int/ageing/publications/lifecourse/alc_lifecourse_training_en.pdf [Accessed 13^th^ Nov, 2019].

[27] Grant MJ, Booth A (2009). A typology of reviews: an analysis of 14 review types and associated methodologies. Health Inf Libr J.

[28] Krlev G, Munscher R and Mulbert K. 2013. Social return on investment (SROI): State-of-the-art and perspectives: a meta-analysis of practice in social return on investment studies published 2000-2012. [Online]. Available at: https://archiv.ub.uni-heidelberg.de/volltextserver/18758/ [Accessed 18^th^ Nov, 2019].

[29] Moher D, Shamseer L, Clarke M, Ghersi D, Liberati A, Petticrew M, Shekelle P, Stewart LA, PRISMA-P Group. Preferred reporting items for systematic review and meta-analysis protocols (PRISMA-P) 2015 statement. Syst Rev. 2015;4(1). 10.1186/2046-4053-4-1 PMC4320440 25554246

[30] Envoy Partnership. 2018. Community Champions. Social return on investment evaluation. [Online]. Available at: https://www.centrallondonccg.nhs.uk/media/92475/Community-Champions-SROI-2018.pdf [Accessed 13th Nov, 2019].

[31] Hanafin S, O’Dwyer K, Creedon M and Mulvaney Clune C. 2018. Social return on investment: PHN-facilitated breastfeeding groups in Ireland. [Online]. Available at: https://www.lenus.ie/handle/10147/623038 [Accessed 13th Nov, 2019].

[32] Arvidson M, Battye F, Salisbury D (2014). The social return on investment in community befriending. Int J Public Sect Manag.

[33] Barnardo’s. 2012. The value of early intervention. Identifying the social return of Barnardo’s Children Centre services. [Online]. Available at: http://www.socialvalueuk.org/app/uploads/2016/03/the_value_of_early_intervention.pdf [Accessed 13th Nov, 2019].

[34] Chance T. 2013. Cambridgeshire’s funded two-year-old childcare social return on investment report. [Online]. Available at: http://www.socialvalueuk.org/app/uploads/2016/04/130624-SROI-Report-CCC-v4-FINAL-1.pdf [Accessed 13th Nov, 2019].

[35] Lakhotia S. 2019. Incredible Years Parenting Programme. Forecast Social Return on Investment Analysis. [Online]. Available at: http://www.socialvalueuk.org/app/uploads/2019/05/Assured-SROI-Report-Incredible-years.pdf [Accessed 13th Nov, 2019].

[36] New Economics Foundation. 2009a. The economic and social return of Action for Children’s Family Intervention Team/5+ Project, Caerphilly. [Online]. Available at: http://www.socialvalueuk.org/app/uploads/2016/03/the_economic_and_social_return_of_action_for_children_s_family_intervention_team5_project_caerphilly.pdf [Accessed 13th Nov, 2019).

[37] New Economics Foundation. 2009c. The economic and social return of Action for Children’s East Dunbartonshire Family Service. [Online]. Available at: http://www.socialvalueuk.org/app/uploads/2016/03/the_economic_and_social_return_of_action_for_childrens_east_dunbartonshire_family_service.pdf [Accessed 13th Nov, 2019].

[38] New Economics Foundation. 2010. The economic and social return of Action for Children’s Family Intervention Project, Northamptonshire. [Online]. Available at: http://www.socialvalueuk.org/app/uploads/2016/03/assurance%20submission%20final%20TVB.pdf [Accessed 13^th^ Nov, 2019].

[39] Action on Addiction. 2014. SROI Analysis. A social return on investment analysis of the M-PACT (Moving Parents And Children Together) Programme. [Online]. Available at: http://www.socialvalueuk.org/app/uploads/2016/03/AOA-SROI-M-PACT-2014.pdf [Accessed 13th Nov, 2019].

[40] Bradly J and Bolas C. 2013. Social return on investment (SROI) of Substance Misuse Work Leicestershire Youth Offending Service. [Online]. Available at: http://www.socialvalueuk.org/app/uploads/2016/03/SROI%20substance%20misuse%20Final%20(1)%20(1).pdf [Accessed 13^th^ Nov, 2019].

[41] Hackett C, Jung Y and Mulvale G. 2017. Pine River Institute: the social return on investment for a residential treatment program. [Online]. Available at: https://static1.squarespace.com/static/555e3952e4b025563eb1c538/t/595252a5d482e9a9a8d855c2/1498567338303/2017+SROI+small.pdf [Accessed 13^th^ Nov, 2019].

[42] New Economics Foundation. 2009b. The economics and social return of Action for Children’s Wheatley Children’s Centre, Doncaster. [Online]. Available at: http://www.socialvalueuk.org/app/uploads/2016/03/the_economic_and_social_return_of_action_for_childrens_wheatley_childrens_centre_doncaster.pdf [Accessed 13th Nov, 2019].

[43] Winrow E and Tudor Edwards R. 2018. Social return on investment of Sistema Cymru-Codi’r To*.* [Online]. Available at: https://cheme.bangor.ac.uk/documents/Codi%27r%20To%20(English)%20.pdf [Accessed 13^th^ Nov, 2019].

[44] Bradly J. 2011. Social return on investment. Evaluation of the Leicestershire and Rutland Community Safer Sex Project. [Online]. Available at: http://www.socialvalueuk.org/app/uploads/2016/03/CSSP_SROI_Evaluation_FINAL.pdf [Accessed 13th Nov, 2019].

[45] Children Our Ultimate Investment. 2010. Social return on investment. COUI. The Teen and Toddlers Programme. [Online]. Available at: http://www.socialvalueuk.org/app/uploads/2016/03/Microsoft_Word_-_COUI_Teens.pdf [Accessed 13th Nov, 2019].

[46] Hoskisson A. 2012. Forecast social return on investment analysis. The Bridge Project Western Australia. [Online]. Available at: http://www.socialvalueuk.org/app/uploads/2016/03/YMCA%20Bridge%20Project%20SROI%20Report%20v2%20(logos%20and%20assurance%20statement).pdf [Accessed 13^th^ Nov, 2019].

[47] Butler W and Leathem K. 2014. A social return on investment evaluation of three ‘Sport for Social Change Network’ programmes in London. [Online]. Available at: https://static1.squarespace.com/static/5733282860b5e9509bc9c4db/t/573de713c2ea51d5e4d8e5c5/1463674646108/Active-Communities-Network-Social-Return-on-Investment-Report.pdf [Accessed 13th Nov, 2019].

[48] Ward F, Thurston M (2009). RESPECT: A Personal Development Programme for Young People at Risk of Social Exclusion: Option One: Social Return on Investment.

[49] Forth Sector Development. 2007. Restart. Social return on investment report. [Online]. Available at: http://www.socialvalueuk.org/app/uploads/2016/04/sroireport-Restart.pdf [Accessed 13th Nov, 2019].

[50] Goodspeed T. 2009. Workwise. Forecast of social return on investment of workwise activities (April 2009 to March 2010). [Online]. Available at: http://www.socialvalueuk.org/app/uploads/2016/04/SROI-Report-Workwise-Oct-09.pdf [Accessed 13th Nov, 2019].

[51] Leck C. 2011. Social return on investment (SROI) Evaluation Report, August 2012 of the Houghton Project (October 2011 to September 2011). [Online]. Available at: http://www.socialvalueuk.org/app/uploads/2016/03/Houghton%20Project%20SROI%20assured.pdf [Accessed 13^th^ Nov, 2019].

[52] NEF Consulting. 2017. Lancashire Wellbeing Service: Social return on investment. Executive summary. [Online]. Available at: https://www.nefconsulting.com/wp-content/uploads/2018/03/LWS-Executive-summary.pdf. [Accessed 13th Nov, 2019].

[53] Gingerbread and New Economics Foundation. 2013. Getting together. The impact of local Gingerbread groups on single parent families in England and Wales. [Online]. Available at: http://www.socialvalueuk.org/app/uploads/2016/03/8468.pdf [Accessed 13th Nov, 2019].

[54] Hoggett J, Ahmed Y, Frost E, Kimberlee R, McCartan K, Solle J and Bristol City Council. 2014. The troubled families programme: A process, impact and social return on investment analysis. [Online]. Available at: http://www.socialvalueuk.org/app/uploads/2016/04/A-Process-Impact-and-SROIA-of-BCC-Troubled-Families-Programme.pdf [Accessed 13th Nov, 2019].

[55] Lukoseviciute L. 2010. Social return on investments in smoking cessation policy in the Netherlands. [Online]. Available at: http://arno.uvt.nl/show.cgi?fid=113851 [Accessed 13^th^ Nov, 2019].

[56] Inge N. 2012. Social return on investment of Ready for Work. [Online]. Available at: http://www.socialvalueuk.org/app/uploads/2016/06/socialreturn.pdf [Accessed 13th Nov, 2019].

[57] Bradly J, Butler W and Leathem K. 2013. A social return on investment (SROI) analysis of Double Impact citywide services in Nottingham for people recovering from alcohol/drug dependence. [Online]. Available at: https://www.doubleimpact.org.uk/wp-content/uploads/2018/05/SROI_report_-_Double_Impact_Aug_2013.pdf [Accessed 13th Nov, 2019].

[58] Bosco A, Schneider J, Broome E (2019). The social value of the arts for care home residents in England: a social return on investment (SROI) analysis of the imagine arts programme. Maturitas..

[59] Health Innovation Network. 2015. Peer support for people with dementia. A social return on investment (SROI) study. [Online]. Available at: https://healthinnovationnetwork.com/wp-content/uploads/2017/01/Social_Return_on_Investment_Study_Dementia_Peer_Support_Groups-1.pdf [Accessed 13th Nov, 2019].

[60] House of Memories. 2014. An evaluation of House of Memories Dementia Training Programme: Midlands Model. [Online]. Available at: https://houseofmemories.co.uk/media/1011/house-of-memories-midlands-evaluation-2014.pdf [Accessed 13th Nov, 2019].

[61] Jones C, Windle G, Tudor ER. Dementia and imagination: a social return on investment analysis framework for art activities for people living with dementia. Gerontologist. 2018:1–12. 10.1093/geront/gny147. 10.1093/geront/gny147 PMC12774629 30476114

[62] Social Value Lab. 2011. Craft Café. Creative Solutions to Isolation and Loneliness. Social return on investment evaluation. [Online]. Available at: http://www.socialvaluelab.org.uk/wp-content/uploads/2013/05/CraftCafeSROI.pdf [Accessed 13th Nov, 2019].

[63] Stay Well at Home. 2012. Stay Well at Home. Social return on investment (SROI) evaluation report – a summary. [Online]. Available at: http://www.socialvalueuk.org/app/uploads/2016/03/ACK-SWaH-report-web1.pdf [Accessed 13th Nov, 2019].

[64] Jones M. 2012. The social value of a community-based health project. Health Living Wessex. Social return on investment report. [Online]. Available at: http://www.socialvalueuk.org/app/uploads/2016/04/HLW_Social_Value_Report_Revised-TVB-Sept12.pdf [Accessed 13th Nov, 2019].

[65] Shipley R and Hamilton L. 2011. Healthwise Hull. Social return on investment – forecast. [Online]. Available at: http://www.socialvalueuk.org/app/uploads/2016/03/SROI%20Report%20-%20Healthwise%20-%20February%202012%20revised%20FINAL.pdf [Accessed 13^th^ Nov, 2019].

[66] Chin C. 2016. Health Disability Sport Partnership: A social return on investment analysis. [Online]. Available at: https://whiasu.publichealthnetwork.cymru/files/6715/0210/7630/The_Health_Disability_Sport_Partnership_-_A_Social_Return_on_Investment_Analysis_FINAL.pdf [Accessed 13th Nov, 2019].

[67] Greenspace Scotland. 2013. Glasgow Health Walks. Social return on investment analysis. 1^st^ April 2011 to 31^st^ March 2012. [Online]. Available at: http://www.socialvalueuk.org/app/uploads/2016/03/Glasgow_Health_Walks_assured%20and%20formatted.pdf [Accessed 13^th^ Nov, 2019].

[68] Lobley N and Carrick K. 2011. Social return on investment evaluation report. Bums off Seats. [Online]. Available at: http://www.socialvalueuk.org/app/uploads/2016/03/BoS%20assured%20version.pdf [Accessed 13^th^ Nov, 2019].

[69] Jones M, Pitt H, Oxford L, Orme J, Gray S, Salmon D, Meand R, Weitkamp E, Kimberlee R and Powell J. 2016. Food for Life. A social return on investment analysis of the locally commissioned programme. Full report. [Online]. Available at: https://www.foodforlife.org.uk/~/media/files/evaluation%20reports/4foodforlifelcssroifullreportv04.pdf [Accessed 13th Nov, 2019].

[70] UK Government. 2012. Public Services (Social Value) Act 2012. [Online]. Available at: http://www.legislation.gov.uk/ukpga/2012/3/enacted [Accessed 12^th^ Nov 2019].

[71] Lang M. 2018. Why social value is so important in the public sector. [Online]. Available at: https://www.supply2govtenders.co.uk/resources/blog/why-social-value-is-so-important-in-the-public-sector/ [Accessed 12^th^ Nov, 2019].

[72] Dynan K and Sheiner L. 2018. GDP as a measure of economic well-being. [Online]. Available at: https://www.brookings.edu/wp-content/uploads/2018/08/WP43-8.23.18.pdf [Accessed 13th Nov, 2019].

[73] Maier F, Schober C, Simsa R, Millner R (2015). SROI as a method for evaluation research: understanding merits and limitations. Voluntas.

[74] Burton-Jeangros C, Cullati S, Sacker A, Blane D, Burton-Jeangros C, Cullati S, Sacker A (2015). xxx. Chapter 1 Introduction. A life course perspective on health trajectories and transitions.

[75] Early Intervention Fund. 2014. Measuring the social impact of Early Intervention initiatives – A Guidance Document. [Online]. Available at: https://can-invest.org.uk/uploads/editor/files/Invest/EIF/Guidance_on_measuring_the_social_impact_of_Early_Intervention_initiatives.pdf [Accessed 18^th^ Nov, 2019].

[76] Coast J (2019). Assessing capability in economic evaluation: a life course approach?. Eur J Health Econ.

[77] Fujiwara D. 2015. The seven principle problems of SROI. [Online]. Available at: http://www.socialvalueuk.org/app/uploads/2016/03/The%20Seven%20Principle%20Problems%20with%20SROI_Daniel%20Fujiwara.pdf [Accessed 12^th^ Nov, 2019].

[78] Jacobson PD, Neumann PJ (2009). A framework to measure the value of public health services. Health Serv Res.

